# Mental disorders are somatic disorders, a comment on M. Stier and T. Schramme

**DOI:** 10.3389/fpsyg.2014.00053

**Published:** 2014-02-07

**Authors:** Marcella Rietschel

**Affiliations:** Department of Genetic Epidemiology in Psychiatry Central Institute of Mental Health, Medical Faculty Mannheim, University of HeidelbergHeidelberg, Germany

**Keywords:** genetics, psychiatry genetics, psychiatry behavior, behavior, disease models

In his paper, Marco Stier argues that mental disorders are irreducible to the brain and cannot be determined in a purely physical manner (Stier, 2013). Instead, he argues that mental disorders can only be determined on the mental level since behavior can only be termed deviant by comparing it to the norms of non-deviant behavior. Thomas Schramme proposes that a psychological conceptualization of mental disorders is necessary since the concept of mental illness is autonomous from somatic medicine and that psychiatry will not find the cure it is looking for through neurobiology (Schramme, 2013).

In the following, I present my personal argument for: (i) why neurobiological research offers the potential for identifying curative therapies for mental disorders despite the lack of a valid explanatory model on the mental level (albeit not to the extent psychiatrists may wish); and (ii) that such research is needed to establish more valid disease models on the mental level. In other words, an understanding of the biological underpinnings of the mental disorders is a prerequisite for a valid disease model. If the definition of deviant behavior requires norms, these norms, and the degree of deviance, will not only depend on the observed behavior *per se* but also on our understanding of its biological underpinnings and its variance. To illustrate this point I will cite two examples. Firstly, phenotypically identical hallucinations are conceptualized differently depending on whether they are the consequence of a high fever or the symptoms of schizophrenia, and whether effective treatment is readily available or not. Secondly, the inability to spell correctly due to dyslexia is judged differently to poor spelling that is attributable to a lack of care or effort (and dyslexia has for the majority of the past 2000 years of our history almost totally escaped diagnosis). Thus, a concept which is not supported by knowledge of the etiological underpinnings can only be partial and, as Hanfried Helmchen points out, potentially dangerous for patients (Helmchen, 2013).

Furthermore, I argue that the concept of “mental disorder” should be abandoned or at least not confined to the so called mental/psychiatric disorders, as in my opinion mental disorders are somatic disorders, and the so-called somatic disorders also associated with varying numbers of psychiatric symptoms.

As a psychiatric geneticist whose research aim is to identify genes involved in the development of psychiatric disorders, I believe that all of the observed clinical symptoms have a biological correlate. The extent to which different societies in different time periods will conceptualize these symptoms as “pathological” will depend on the society in question. Factors such as the severity of the individual's suffering (e.g., paranoia, anxiety) or the degree of severity attributed to given symptoms by society (e.g., obesity, gambling, sexual deviations) will play a role. Nevertheless the biological underpinnings of deviations from the so-called “normal” will, in many cases, be identifiable. Although I personally consider it unlikely that science will ever provide an exclusively neurophysiological explanation of mental disorders, I am convinced that diagnosis will eventually be based upon assessment of the physiology of the individual patient.

At present however, no such biology-guided diagnoses exist. Although research has established that mental disorders are complex and that their development involves interactions between genetic and environmental factors, their etiology remains largely unknown. Current psychiatric classification systems, such as DSM and ICD, define psychiatric disorders as distinct disease entities. According to these diagnostic systems, a diagnosis should be assigned when a given number of symptoms have been present over a specified period of time. Despite high diagnostic reliability between psychiatrists and evidence from family studies that relatives of index patients have an increased risk of being assigned the same diagnosis, the clinical presentation of psychiatric disorders differs widely between patients, and diverse courses and outcomes are observed within diagnostic categories. Furthermore, no single clinical symptom is either pathognomonic of, or necessary for, a given psychiatric diagnosis, and considerable symptom-overlap is observed between diagnostic categories.

As, in contrast to somatic disorders, no objective laboratory measures are yet available to refine psychiatric diagnosis, the establishment of a diagnostic system that is biologically based will require a more comprehensive knowledge of the etiology and pathophysiology of psychiatric disorders and/or their presenting symptoms.

Since Griesinger, biological psychiatry has conceptualized psychiatric illnesses as disorders of the brain. However, other brain disorders such as migraine, epilepsy, and neoplasms are generally treated by neurologists rather than psychiatrists. In cases where a causal biological reason for psychotic or depressive symptoms has been identified, the disorder ceases to be a mental disease in the strict sense of the term. This is exemplified by endocrine conditions (e.g., porphyria, hyper-, and hypothyroidism); metabolic conditions (e.g., hypoglycaemia); hepatic, renal, or autoimmune conditions; and viral infections. Indeed a DSM criterion for assigning a diagnosis of schizophrenia is that *“The disturbance is not attributable to the physiological effects of a substance or another medical condition.”* Similarly, a DSM diagnosis of psychotic disorder requires that “*no specific and direct causative physiological mechanisms associated with a medical condition can be demonstrated.”*

Recent findings in psychiatric genetics may provide insights into how mental disorder should be conceptualized. For many years, whole genome screening and the process of relating millions of genetic variants with a complex disorder while taking into account environmental factors and personal life-experiences were considered impossible. However, these processes are now available to researchers.

In recent decades, extensive efforts have been made to identify susceptibility factors for psychiatric disorders such as schizophrenia, bipolar disorder, autism, attention deficit hyperactivity disorder, major depression, and alcohol dependence. The results of formal genetic investigations, e.g., family, twin, and adoption studies, have provided unequivocal evidence that environmental factors as well as inherited genetic variation play a substantial role in the etiology of these disorders. Heritability estimates suggest that genetic factors account for 75–80% of the variability observed in susceptibility to schizophrenia, bipolar disorder, autism and attention deficit hyperactivity disorder and 35–50% of that for alcohol addiction and major depression. For other common complex disorders, such as diabetes, breast cancer, and Crohn's disease, heritability ranges between 55 and 70% (Sullivan et al., 2012). Thus, the contribution of genetic factors to schizophrenia, bipolar disorder, and autism is relatively high. Schizophrenia is also one of the complex common disorders that account for the majority of genome-wide significant findings identified in genome-wide association studies (GWAS) since their introduction in less than a decade ago.

Recent calculations indicate that around 6000–10,000 common variant are involved in the etiology of schizophrenia, and that these variants are not confined to genes expressed in the central nervous system (Ripke et al., 2013). Rather, these variants are located across all chromosomes. It is therefore very likely that the influence of these variants is not restricted to the brain. Thus, a given variant could act as a risk factor for both, a mental as well as a somatic disorder. Many somatic and mental disorders display co-morbidity, and the question therefore arises as to whether these conditions should be viewed as two separate diseases, or whether such states represent a single disease with somatic as well as mental manifestations. Formal genetic studies of co-morbidity between cardiovascular disease and depressive disorders, e.g., suggest the latter. Furthermore, initial molecular genetic studies suggest that stress, for example, is a risk factor for both depression and cardiovascular disease: the influence of a major risk gene (FTO) for obesity is particularly pronounced in depressed persons, and genetic variation in the” stress gene” NPY modifies weight gain under conditions of stress.

Interestingly, among the most significant findings for schizophrenia are variants located in the Major Histocompatibility Complex Region, a locus which hosts, among others, genes responsible for immune reactions. This evidence underlines the finding of formal genetic and candidate gene studies that genetic as well as environmental factors contribute to these disorders.

I argue that all mental disorders are somatic disorders, and that what we currently term mental disorders are actually somatic disorders for which the somatic component is too weak to be detected. That is, due to the high sensitivity of the human brain, and the extreme level of functioning demanded of it in modern life, even harmless somatic changes may have a detrimental influence on brain function.

An example may serve to illustrate this point. Genetic and biochemical studies indicate that immunological processes play an important role in depression, or at least in a subset of them. Viral infections such as a common cold in turn also involve immunological reactions and can present with all of the symptoms required to assign a diagnosis of MD (e.g., markedly diminished interest or pleasure in almost all activities, fatigue, diminished ability to concentrate, loss of appetite, hypersomnia). If such an infection escapes diagnosis it is possible that the patients will receive the diagnosis of depression. I personally have seen patients who had been assigned a diagnosis of depression and who actually had suffered from unrecognized infections such as borreliose and hepatitis. New infectious agents are identified each year, and it is possible that a proportion of patients who are diagnosed with depression today are being misdiagnosed since the causal agent has not yet been identified. But the question is whether this really should be considered a simple misdiagnosis.

While I would argue that all mental disorders are in fact somatic disorders (including the brain as an organ), this implies neither that the cause must originate in the soma, nor that conclusions may be drawn concerning the optimal mode of treatment.

Numerous studies have shown that factors, such as poverty, stressful life events, and child abuse, are major risk factors for depression (although the effect of a given environmental factor can differ substantially depending on the genetic make-up of the individual). Furthermore, the environment can have long lasting effects in terms of which genes will be expressed. This in turn will influence how that particular individual will respond to a future environmental stimulus. A decade ago, research in rats demonstrated for the first time that post-natal maternal care could influence stress hormone receptors, and that this predisposed the affected animal to more pronounced reactions to stress in later life (Szyf et al., 2005). These findings were subsequently replicated in humans. Furthermore, a human genome-wide methylation study by our group revealed that prenatal maternal stress impacted on the methylation pattern in the newborn. These results were then replicated in studies of monkeys and rats. Thus, *environmental* factors can impact on mental well-being through the soma, and can therefore predispose to further reactions to environmental factors through *somatic* signatures. Further knowledge is needed to define whether the optimal therapy for the resulting depression should be delivered on a direct somatic level or through psychotherapy.

To conclude: I argue firstly, that all mental disorders are somatic disorders, and that somatic disorders present with varying proportions of somatic and/or mental symptoms; and secondly, that both the identification of the underlying genetic and environmental factors as well as optimal causal therapies is important and feasible. This neurobiological approach is promising even in the absence of a valid disease model on the mental level and will in turn inform such a model. Independent of current disease models, it is important for therapists to remember that patients suffer from their symptoms *per se* rather than from the underlying causes of these symptoms. Thus, the management of patients who display mental symptoms—with or without somatic symptoms—requires both an understanding of the nature and subjective impact of these symptoms and appropriate therapeutic empathy.

## References

[B1] Helmchen H. (2013). Different conceptions of mental illness: consequences for the association with patients. Front. Psychol. 4:269 10.3389/fpsyg.2013.00269 23720646 PMC3654202

[B1a] Ripke S. O'Dushlaine C. Chambert K. Moran J. L. Kähler A. K. Akterin S. (2013). Genome-wide association analysis identifies 13 new risk loci for schizophrenia. Nat. Genet. 45, 1150–1159 10.1038/ng.2742 23974872 PMC3827979

[B2] Schramme T. (2013). On the autonomy of the concept of disease in psychiatry. Front. Psychol. 4:457 10.3389/fpsyg.2013.00457 23882252 PMC3715727

[B3] Stier M. (2013). Normative preconditions for the assessment of mental disorder. Front. Psychol. 4:611 10.3389/fpsyg.2013.00611 24058357 PMC3766858

[B3a] Sullivan P. Daly M. J. Ot'Donovan M. (2012). Genetic architectures of psychiatric disorders: the emerging picture and its implications. Nat. Rev. Genet. 13, 537–551 10.1038/nrg3240 22777127 PMC4110909

[B3b] Szyf M. Weaver I. C. Champagne F. A. Diorio J. Meaney M. J. (2005). Maternal programming of steroid receptor expression and phenotype through DNA methylation in the rat. Front. Neuroendocrinol. 26, 139–136 10.1016/j.yfrne.2005.10.002 16303171

